# The Role of Catalase in Pulmonary Fibrosis

**DOI:** 10.1186/1465-9921-11-183

**Published:** 2010-12-29

**Authors:** Nao Odajima, Tomoko Betsuyaku, Katsura Nagai, Chinatsu Moriyama, Da-Hong Wang, Tomoko Takigawa, Keiki Ogino, Masaharu Nishimura

**Affiliations:** 1 First Department of Medicine, Hokkaido University School of Medicine, N-15, W-7, Kita-ku, Sapporo 060-8638, Japan; 2 Department of Public Health, Okayama University Graduate School of Medicine, 2-5-1 Shikata-chou, Okayama 700-8558, Japan

## Abstract

**Background:**

Catalase is preferentially expressed in bronchiolar and alveolar epithelial cells, and acts as an endogenous antioxidant enzyme in normal lungs. We thus postulated epithelial damage would be associated with a functional deficiency of catalase during the development of lung fibrosis.

**Methods:**

The present study evaluates the expression of catalase mRNA and protein in human interstitial pneumonias and in mouse bleomycin-induced lung injury. We examined the degree of bleomycin-induced inflammation and fibrosis in the mice with lowered catalase activity.

**Results:**

In humans, catalase was decreased at the levels of activity, protein content and mRNA expression in fibrotic lungs (n = 12) compared to control lungs (n = 10). Immunohistochemistry revealed a decrease in catalase in bronchiolar epithelium and abnormal re-epithelialization in fibrotic areas. In C57BL/6J mice, catalase activity was suppressed along with downregulation of catalase mRNA in whole lung homogenates after bleomycin administration. In acatalasemic mice, neutrophilic inflammation was prolonged until 14 days, and there was a higher degree of lung fibrosis in association with a higher level of transforming growth factor-β expression and total collagen content following bleomycin treatment compared to wild-type mice.

**Conclusions:**

Taken together, these findings demonstrate diminished catalase expression and activity in human pulmonary fibrosis and suggest the protective role of catalase against bleomycin-induced inflammation and subsequent fibrosis.

## Background

Pulmonary fibrosis is a chronic interstitial lung disease resulting from damage to the lung parenchyma by varying patterns of inflammation and fibrosis with a high mortality rate and poor response to available medical therapy [1]. An imbalance of oxidants and antioxidants can alter a number of processes thought to contribute to the pathogenesis of pulmonary fibrosis, such as activation of redox-sensitive signaling pathways and transcription factors, modification of immune function, modulation of the protease/antiprotease balance, and activation of fibroblasts [2-4]. It is well known that accumulated inflammatory cells such as neutrophils, which release toxic oxidants, are also capable of inducing oxidant-mediated lung parenchymal cell toxicity in the process of fibrosis [4].

Catalase, a 240-kD tetrameric heme protein, is one of the major intracellular antioxidant enzyme responsible for detoxifying the hydrogen peroxide produced under physiological conditions to oxygen and water [5]. Excessive hydrogen peroxide is harmful to almost all cell components, and thus its rapid and efficient removal is vitally important for aerobic organisms [6]. Further to this idea, in one study a transgenic mouse overexpressing catalase localized to mitochondria showed an extended life span due to enhanced protection of mitochondria from reactive oxygen species (ROS), in which catalase overexpression also suppressed age-related DNA oxidation in skeletal muscle [7]. It has been known that damage to the mitochondrial membrane by ROS leads to a loss in membrane potential and pore-opening, causing swelling, leakage of cytochrome c, and initiation of apoptosis [8]. Arita et al. recently reported that targeting of catalase directly to the mitochondria in lung epithelial cells protected the cells from hydrogen peroxide-induced apoptosis [9].

In the lungs, catalase is expressed during the later stages of development, is constitutively expressed in airway and alveolar epithelial cells and in macrophages [10-12], and plays an important role in the endogenous antioxidant defense system. Studies are limited regarding the role of catalase in pulmonary fibrosis in humans [3,13], although catalase was found to be decreased in airway epithelium exposed to 100% O_2 _[14], in lung cancer [15], and in asthma [16]. The regulatory mechanisms and role of catalase in the development of pulmonary fibrosis have largely remained to be determined.

We thus hypothesized that (A) catalase is diminished in human pulmonary fibrosis and in mouse bleomycin-induced lung injury, (B) a decrease in catalase particularly occurs in bronchiolar epithelial cells and/or in various types of abnormal re-epithelialization in fibrotic lungs, and finally (C) the deficiency in catalase activity in the lungs results in predisposing the lung to worsening lung inflammation and subsequent fibrosis. In this study, we found catalase has a protective role in the lung fibrosis.

## Materials and methods

### Patients and tissue collection

The study population comprised 12 patients with pulmonary fibrosis. Appling the diagnostic criteria of the American Thoracic Society/European Respiratory Society (ATS/ERS) international multidisciplinary consensus classification [1], each diagnosis was based on the standard clinical criteria and histopathological analyses of lung tissues obtained by video-assisted thoracoscopy-guided lung biopsy or surgical lobectomy as previously described [17]. All control lung specimens were obtained from 10 patients who had never smoked and who underwent lung lobectomy for small peripheral tumors. Immediately after biopsy or lobar resection, tissues were frozen as soon as possible before RNA and protein extraction or were fixed in 10% neutral buffered formalin for immunohistochemistry as previously described [18]. Written informed consent to participate in the study was obtained from all patients, and the Ethics Committee of Hokkaido University School of Medicine approved the study. Table 1 summarizes the clinical characteristics of the control subjects and patients with pulmonary fibrosis. The mean interval between the onset of symptoms and pathological diagnosis was 19.8 months. Neither the patients nor control subjects had received any drugs which might cause drug-induced pneumonitis at the time of this study.

**Table 1:** Clinical Characteristic of Control and Pulmonary Fibrosis Patients

	control	Pulmonary Fibrosis
Number of subjects, female/male	10, 7/3	12, 8/4
Age, yr	66 ± 4	63 ± 2
Cigarette smoking, never/former/current	10/0/0	7/1/4
Pathological diagnosis	NA	3 UIP
		9 NSIP
VC, % pred	109 ± 5	88 ± 6
FEV1/FVC, %	76 ± 1	80 ± 2
DL_CO_, % pred	103 ± 7	65 ± 5
PaO2, torr	87 ± 3	77 ± 4
Collagen vascular disease	0	3 Sjs

(Mean ± SE)

UIP, usual interstitial pneumonia (*: p < 0.05 vs. control)

NSIP, nonspecific interstitial pneumonia

Sjs, Sjögren's syndrome

### Animals and experimental protocols

Male C57BL/6J mice (6-8 weeks old) were purchased from CLEA Japan (Tokyo, Japan). The mice were housed in plastic cages under a 12-h light/dark cycle, fed standard chow, and given free access to food and water. Male wild-type mice (C3H/AnLCsaCsa) and male homozygous acatalasemic mutant mice (C3H/AnLCsbCsb) at the age of 15 weeks were used [19]. After an intraperitoneal injection of ketamine and xylazine for sedation and anesthesia, 0.05 U of bleomycin (Blenoxane; Nippon Kayaku, Tokyo, Japan) was intratracheally administrated as described [20]. After 7, 14, and 21 days, the animals were killed and their lungs were processed as described below. Mice that had not undergone manipulation served as controls. All experimental protocols and procedures were approved by the Ethics Committee on Animal Research of the Hokkaido University School of Medicine.

### Bronchoalveolar lavage (BAL) and sampling of mouse lung tissue

Mice were sacrificed by CO_2 _narcosis, and then the lungs were lavaged with 0.6 ml of saline three times through a tracheal cannula. Total cell counts and cell differentials in the BAL fluid were determined as described [20]. After BAL was performed, the lungs were fixed by inflation with 10% buffered formalin (Mildform 10N; Wako Pure Chemical Industries, Osaka, Japan) at a constant pressure of 25 cm H_2_O and embedded in paraffin for morphometric assessment, or inflated with diluted Tissue-Tek OCT (Sakura Finetek U.S.A., Torrance, CA, USA) (50% vol/vol in RNase-free PBS containing 10% sucrose), and then stored frozen at -80°C for RNA and protein extraction as previously described [21,22].

### Morphometric assessment

Four mid-sagittal sections of the lungs (4 μm) were stained with hematoxylin and eosin. An observer with no prior knowledge of the animal group assignment assessed 30 randomly chosen regions per tissue sample at a magnification of ×100 and determined the average score of fibrosis. The severity of fibrosis was semiquantitatively assessed using Ashcroft score, as previously described [23,24]. Briefly, the grade of lung fibrosis was scored on a scale of 0 to 8 as follows: grade 0, normal lung; grade 1, minimal fibrous thickening of alveolar or bronchial walls; grade 3, moderate thickening of walls without obvious damage to the lung architecture; grade 5, increased fibrosis with definite damage to the lung structure and formation to fibrous bands or small fibrous masses; grade 7, severe distortion of structure and large fibrous areas; grade 8, total fibrous obliteration of the field. If there was any difficulty in deciding between two odd-numbered categories, the field would be given the intervening even-numbered score. Alveolar bronchiolization was identified as cells resembling bronchiolar epithelium lining normal or thickened alveolar walls, often in an acinar formation, and was graded from 1 to 3 as previously described [20]. The composite bronchiolization score was calculated as the incidence of bronchiolization multiplied by each grade and summed up in each animal.

### Immunohistochemistry

Catalase immunohistochemistry was performed using a CSA kit (DAKO Japan, Kyoto, Japan) according to the manufacturer's protocol. Tissue sections were incubated with a rabbit anti-catalase antibody (Calbiochem-Novabiochem, San Diego, CA, USA) diluted 1:10,000 at room temperature for 1 hour. The sections were counterstained with hematoxylin. To avoid inter-run variations in immunoreactions, all specimens were stained in the same run using identical reagents. Staining of alveolar macrophages served as the internal positive control for catalase. Rabbit serum was used for negative controls.

### Laser capture microdissection (LCM) of bronchiolar epithelial cells in mouse lung

Bronchiolar epithelial cells were selectively obtained from the lungs by LCM using a PixCell II System (Arcturus Engineering, Mountain View, CA, USA). Bronchiolar epithelial cells were retrieved from the junction of the terminal bronchioles and alveolar ducts and proximally along airways of up to ~250 μm in diameter, as described [22,25].

### Quantitative reverse transcriptase-polymerase chain reaction (RT-PCR)

Total RNA was extracted using the RNeasy^® ^Mini kit (Qiagen, Hilden, Germany) from lung tissue homogenates or LCM-retrieved bronchiolar epithelial cells. Complementary DNA templates were synthesized using RT (Applied Biosystems, Foster City, CA, USA) and mRNA levels were quantified by a 5'-exonuclease based fluorogenic PCR using a 7300 Real Time PCR System (Applied Biosystems), as described [22,25], with TAKARA master mix (TAKARA BIO INC, Shiga, Japan) according to the manufacturer's instructions. The TaqMan Gene Expression Assays probes^® ^were Hs00156308_m1 for human catalase, Mm00437992_m1 for murine catalase, Mm01178820_m1 for murine transforming growth factor-β (TGF-β), Mm00802331_m1 for murine collagen III, Mm00433659_m1 for CXCL1/KC (keratinocyte-derived chemokine), Mm 00434228_m1 for murine interleukin-1β (IL-1β), and Mm 00436450_m1 for murine CXCL2/MIP-2 (macrophage inflammatory protein-2) (Applied Biosystems), and the levels were normalized against glyceraldehyde-3-phosphatase-dehydrogenase (GAPDH) mRNA (human) or β2-microglobulin (β2 MG) mRNA (mouse). In some experiments, 18S rRNA (Ribosomal RNA control reagents^®^) or β-glucuronidase (BGUS) (Mm 00446953_m1) were used for normalization.

### Western blotting

Frozen lung tissues were homogenized and the samples were prepared as previously described [18]. The samples (10 μg of protein) were resolved by electrophoresis under reducing conditions and transferred to Immun-Blot PVDF membranes (Bio-Rad Laboratories, Hercules, CA, USA). The membranes were then incubated overnight at 4°C with rabbit anti-catalase antibody (Calbiochem-Novabiochem) diluted 1:4,000 followed by horseradish peroxidase-conjugated anti-rabbit immunoglobulin (DAKO Japan) diluted 1:20,000. Because the use of β-actin as a normalizing control is limited in human lung diseases [26], loading homogeneity was determined based on an equal amount of total protein in each sample.

### Lung catalase and glutathione peroxidase activity

Frozen lung tissues were homogenized in lysis buffer and used for assessment of the activities of calatase and glutathione peroxidase using commercially available kits, according to the manufacturer's protocol (Cayman Chemical, Ann Arbor, Michigan, USA). Catalase activity was determined based on the reaction of the enzyme with methanol in the presence of an optimal concentration of hydrogen peroxide. The enzyme reaction of glutathione peroxidase was monitored by adding tert-butyl hydroperoxide as a substrate in the presence of glutathione, glutathione reductase and nicotinamide adenine dinucleotide phosphatase.

### Measurement of collagen content of the lung

Collagen content of the lung was determined by assaying soluble collagen using the Sircol Collagen Assay kit (Biocolor, Belfast, Northern Ireland), according to the manufacturer's instructions.

### Assessment of protein carbonyls

Carbonylation of BALF proteins was assessed, as described previously [27,28]. Briefly, 16 μl of unconcentrated BALF was derivatized with dinitrophenylhydrazine (DNP) using the OxyBlot Protein Oxidation Detection Kit (Chemicon International, Temecula, CA) and was separated by electrophoresis on 10% SDS-polyacrylamide electrophoresis gels. Western blots were performed using anti-DNP antibody, followed by scanning with a GT-9500 scanner (Epson, Nagano, Japan); the intensity of the bands was calculated using NIH Image software (version 1.62). On each blot, the recorded total DNP intensity of all bands detected in each lane or bands detected for the same molecular weight was divided by that of a standard sample. The carbonyl content is given in terms of Arbitrary Units (AU)."

### Statistical analysis

Results are expressed as mean ± SEM. The statistical significance of the values at each time point after bleomycin treatment was evaluated by Kruskal-Wallis test. Mann-Whitney U test was applied to comparisons between two groups in the mouse and human studies. Differences were considered significant at p < 0.05 (StatView J 5.0, SAS Institute Inc., Cary, NC, USA).

## Results

### Catalase is decreased in human pulmonary fibrosis

**Figure 1:**
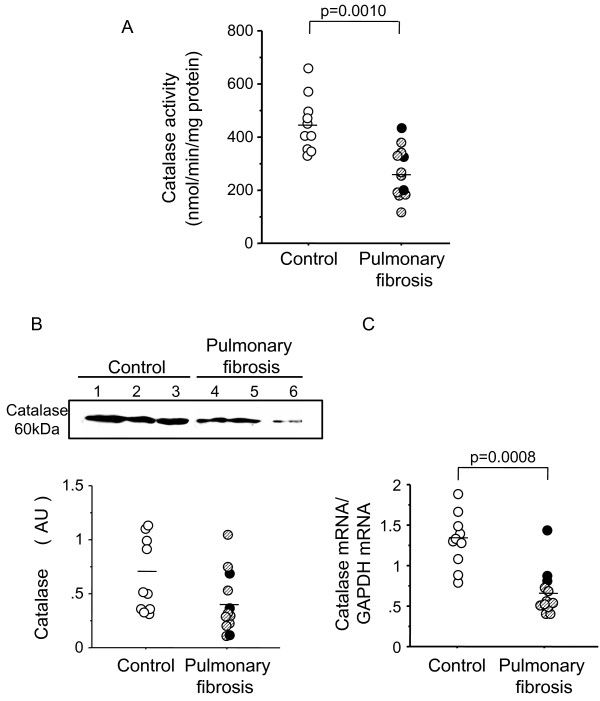
**Catalase in human lung tissue**. Lung catalase is decreased in pulmonary fibrosis than in controls. (A) Catalase activity. (B) Western blotting. Lanes 1-3, control subjects; lanes 4, 5, pulmonary fibrosis patients with NSIP; lane 6, pulmonary fibrosis patients with UIP. (C) Catalase mRNA. Black and hatched circles indicate the subjects who were pathologically diagnosed as UIP and NSIP, respectively, among pulmonary fibrosis patients. GAPDH, glyceraldehyde-3-phosphatase-dehydrogenase; AU, arbitrary units

**Figure 2:**
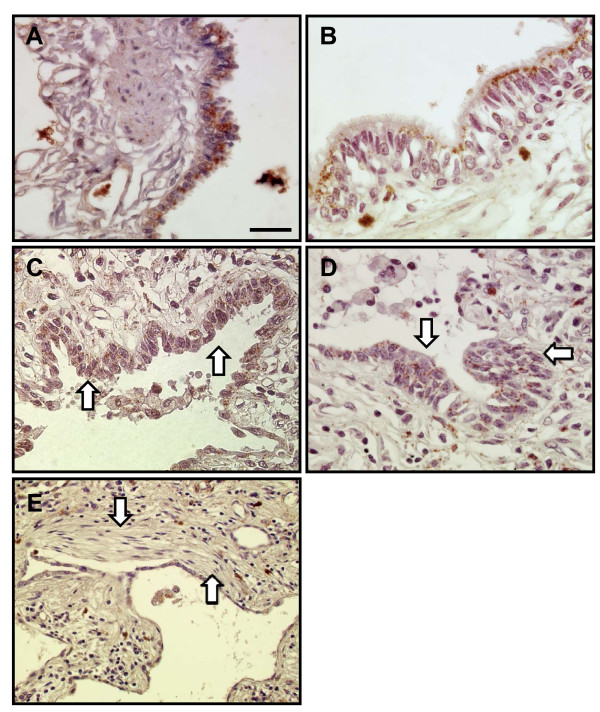
**Immunohistochemical localization of catalase in human lung**. Bronchiolar epithelium in normal control lung shows strong staining (A), whereas bronchiolar epithelium in fibrotic area shows weak staining (B). Bronchiolization (C: white arrows) and squamous metaplasia (D: white arrows) shows faint staining and fibroblastic foci shows negative staining (E: white arrows). Scale bar = 25 μm.

We first assessed whether the catalase activity is altered in human fibrotic lungs. The levels of catalase activity in lung tissue were significantly lower in pulmonary fibrosis compared with controls (p = 0.0010), without any obvious difference between UIP and NSIP (318.8 ± 67.6 nmol/min/mg protein vs. 249.0 ± 29.5; NS) (Figure 1A). To assess whether the reduction in catalase activity in fibrotic lungs is due to the decreased synthesis of catalase, we quantified catalase expression in lung tissues using Western blotting and quantitative RT-PCR. The level of catalase protein in the fibrotic lungs tended to be lower than in the control lungs (p = 0.0559) (Figure 1B). The level of lung tissue catalase mRNA was significantly lower in the fibrotic lungs than control lungs (p = 0.0008) (Figure 1C). The significance of catalase mRNA expression between the two groups persisted when normalized by 18s rRNA (0.62 ± 0.1 vs. 1.6 ± 0.1, p = 0.0002). These results show that the diminished catalase activity in the fibrotic lungs is associated with catalase downregulation at the protein and mRNA levels, although it should be noted that this outcome is also related to a difference in the cellularity of homogenized lung tissues between control and fibrotic lungs. Immunohistochemistry was then performed to localize catalase in fibrotic lungs. Catalase was predominantly localized in bronchiolar epithelial cells (Figure 2A) as well as in type II epithelial cells and alveolar macrophages in control lungs. This was in line with findings by Kaarteenaho-Wiik and Kinnula [12]. In contrast, bronchiolar epithelial cells in fibrotic lungs showed decreased catalase expression to various degrees (Figure 2B). Abnormal re-epithelialization, such as bronchiolization (Figure 2C) and squamous metaplasia (Figure 2D), were barely stained for catalase. Fibroblastic foci were exclusively negative for catalase (Figure 2E).

### Contamination by red blood cells does not contribute to catalase activity of the lungs

Because high catalase levels are found in erythrocytes [29], we removed residual blood by perfusing lungs with saline and compared catalase activity between perfused and unperfused lungs. The catalase activity of saline-perfused lungs was not statistically different from unperfused lungs (252.0 ± 21.4 nmol/min/mg protein vs. 189.7 ± 27.7, NS), suggesting that catalase activity in lung homogenates is not due to circulating erythrocytes, but rather originates from lung structural cells.

### Catalase is decreased in bleomycin-induced lung fibrosis in C57BL/6J mice

**Figure 3:**
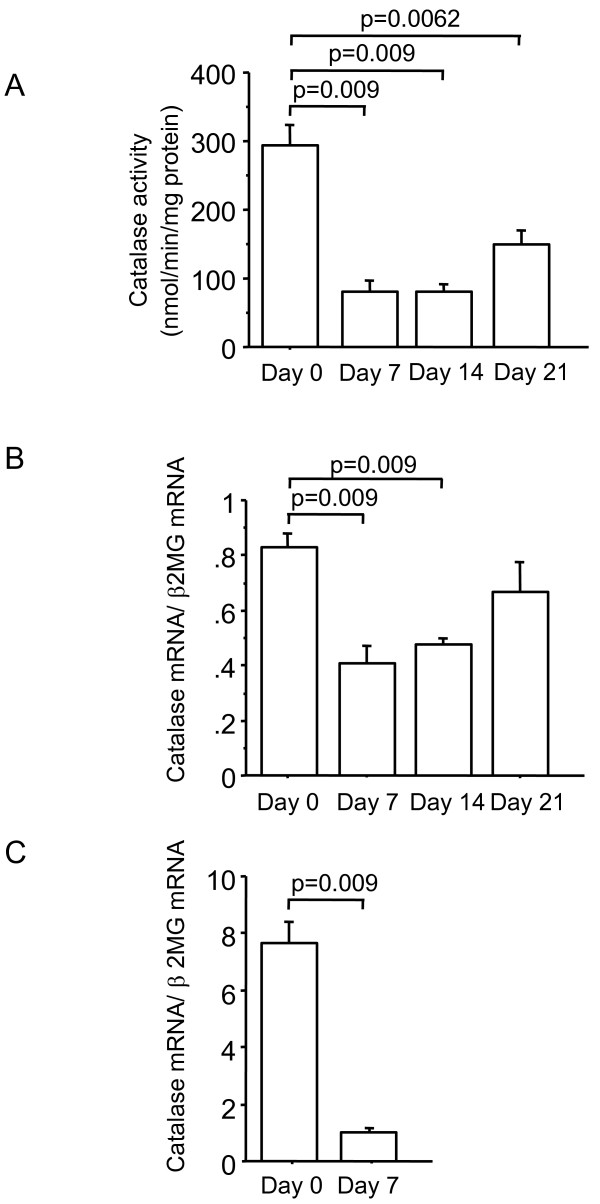
**Lung Catalase in C57BL/6J mice**. Administration of bleomycin decreases lung catalase. (A) Catalase activity. (B) Catalase mRNA. (C) Catalase mRNA in mouse bronchiolar epithelial cells retrieved using LCM. β2MG, β2-microglobulin. Day 0; untreated, Day 7; 7 days after bleomycin administration, Day 14; 14 days after bleomycin administration, Day 21; 21 days after bleomycin administration.

Several studies have demonstrated that bleomycin administration decreases the antioxidant capacity in lung tissue, which aggravates pulmonary fibrosis [30,31]. In order to investigate whether catalase activity and mRNA are also decreased during the development of lung fibrosis, C57BL/6J mice were subjected to intratracheal administration of bleomycin. The levels of catalase activity in whole lung homogenates were significantly lower at 7, 14, and 21 days after intratracheal bleomycin administration compared with untreated controls (p < 0.01) (Figure 3A), which is in line with the findings of previous studies [32,33]. Whole lung catalase mRNA expression was significantly decreased at 7 and 14 days after intratracheal bleomycin administration compared with controls (p < 0.01, respectively) (Figure 3B). The significance of catalase mRNA expression among the groups persisted at 7 and 14 days when normalized by BGUS (p < 0.05, respectively). The data suggest that catalase is downregulated at transcriptional levels, resulting in impaired catalase activity in bleomycin-induced lung fibrosis in mice, as was seen in human IP lungs. We observed that catalase is predominantly expressed in bronchiolar epithelium in normal lungs, and is diminished in IP lungs, especially in bronchiolar epithelium and in abnormal re-epithelialization, such as bronchiolization and squamous metaplasia in humans (Figure 2). We then examined the dynamic change in bronchiolar catalase expression following administration of bleomycin in mice. Using LCM we harvested bronchiolar epithelial cells from lungs in order to quantify catalase mRNA expression *in vivo*, as previously described [18]. Catalase mRNA was present in bronchiolar epithelial cells, and the expression levels were significantly lower at 7 days after bleomycin administration compared with untreated lungs (p = 0.009) (Figure 3C).

### No compensatory increase in glutathione peroxidase activity was observed for catalase in bleomycin-treated acatalasemic mice

**Figure 4:**
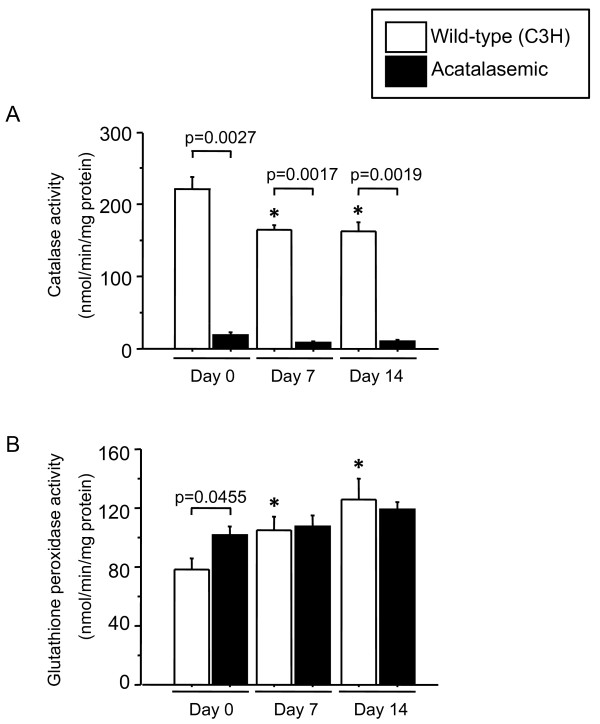
**Changes in catalase and glutathione peroxidase activities in wild-type and acatalasemic mice**. (A) Lung catalase activity is decreased after bleomycin administration in wild-type (C3H/AnLCsaCsa), whereas it is less than 10% in acatalasemic mice at any time point. (B) In the lungs of untreated acatalasemic mice glutathione peroxidase activity is higher compared with wild-type mice, although no further increase is observed following bleomycin administration. Day 0; untreated, Day 7; 7 days after bleomycin administration, Day 14; 14 days after bleomycin administration. *; p < 0.05 vs. Day 0.

To investigate the consequence of decreased catalase activity in the lung during the development of fibrosis, we used acatalasemic mice (C3H/AnLCsbCsb). The untreated lungs of acatalasemic mice possess only 8.1% of catalase activity compared with those of wild-type mice (C3H/AnLCsaCsa) (Figure 4A), although acatalasemic mice demonstrate equivalent levels of catalase mRNA compared with wild-type mice (0.9 ± 0.1 vs. 0.6 ± 0.1, NS). The lung catalase activity in wild-type mice continued to decrease until 14 days following bleomycin administration (Figure 4A), which was consistent with the findings in C57BL/6J mice shown in Figure 3A. The catalase activity remained markedly lower in acatalasemic mice compared to wild-type mice at any time point following bleomycin administration (p < 0.01, respectively) (Figure 4A). Catalase and glutathione peroxidase are the two major enzymes physiologically involved in the detoxification of hydrogen peroxide, and thus protect tissue from oxidant-mediated injury. Therefore we next examined whether glutathione peroxidase could compensate for catalase. Untreated acatalasemic mice had higher glutathione peroxidase activity in the lungs compared with wild-type mice, although no further increase in glutathione peroxidase activity was observed in the lungs of acatalasemic mice following bleomycin administration (Figure 4B). On the other hand, glutathione peroxidase activity was significantly increased at 7 and 14 days in wild-type mice along with a decrease in lung catalase activity. These data suggest a difference in the compensatory mechanism of glutathione peroxidase between wild-type and acatalasemic mice.

### Acatalasemia sensitizes bleomycin induced-inflammation and prolongs bleomycin induced-upregulation of proinflammatory cytokines

We then used the acatalasemic mice to address how the deficiency in catalase activity affected the lung inflammation induced by bleomycin. In BAL fluid, total numbers of inflammatory cells were increased after bleomycin administration in both types of mice. However, the elevations of total cell numbers, lymphocytes and neutrophils were prolonged in acatalasemic mice compared with wild-type mice and showed significant difference at 14 days between wild-type mice and acatalasemic mice (Table 2), suggesting sustained inflammation in acatalasemic mice after bleomycin administration.

**Table 2:** Bronchoalveolar Lavage Fluids in Wild-type and Acatalasemic Mice

	Total cells (× 10^4^/ml)	Cell differentials (× 10^4^/ml)		
		
		Macrophages	Lymphocytes	Neutrophils
**Wild-type (C3H)**				
Day 0 (n = 6)	1.4 ± 0.5	1.3 ± 0.5	0.1 ± 0	0 ± 0
Day 7 (n = 7)	17.8 ± 7.0*	10.7 ± 4.8	2.6 ± 1.3	4.3 ± 2.0*
Day 14 (n = 12)	8.7 ± 1.1*	5.9 ± 0.7	1.7 ± 0.3	1.1 ± 0.4*
**Acatalasemic**				
Day 0 (n = 7)	1.5 ± 0.4	1.3 ± 0.3	0.2 ± 0.1	0 ± 0
Day 7 (n = 7)	6.5 ± 0.9*	4.6 ± 0.6*	1.1 ± 0.2*	0.8 ± 0.2*
Day 14 (n = 10)	13.5 ± 1.5*^†^	7.6 ± 1.1*	3.2 ± 0.4*^†^	2.8 ± 0.7*^†^

*: p < 0.05 vs. Day 0 (Mean ± SE)

†: p < 0.05 vs. Wild-type mice (C3H/AnLCsaCsa)

**Figure 5:**
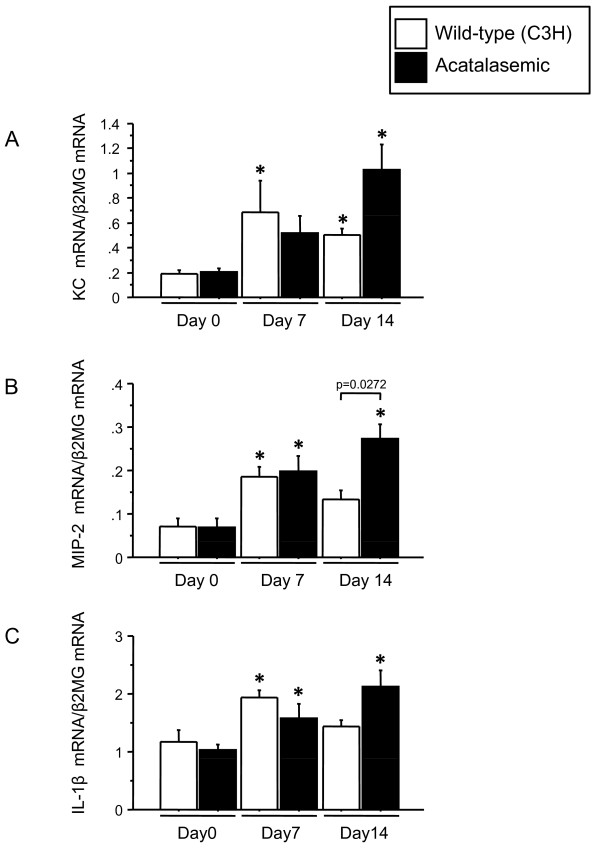
**Changes in expression of inflammatory cytokines in wild-type and acatalasemic mice**. Upregulation of proinflammatory cytokines are sustained in acatalasemic mice (A) KC (B) MIP-2 (C) IL-1β. β2MG, β2-microglobulin. *; p < 0.05 vs. Day 0. Day 0; untreated, Day 7; 7 days after bleomycin administration, Day 14; 14 days after bleomycin administration.

In bleomycin-induced lung injury animal models, inflammatory cytokines have been reported to be temporarily increased in the lungs [34]. In order to elucidate the mechanism of sustained inflammation in acatalasemic mice following bleomycin administration, we quantified the levels KC, MIP-2 and IL-1β expression in whole lung homogenates. KC mRNA was elevated at 7 days after bleomycin administration in both types of mice, but acatalasemic mice showed further elevation at 14 days (Figure 5A). These tendencies were also found for MIP-2 mRNA and IL-1β mRNA (Figure 5B, C). Sustained upregulation of these proinflammatory cytokines in acatalasemic mice may, at least in part, explain the elevation in neutrophils even at 14 days after bleomycin administration.

### Acatalasemia accelerates fibrosis and bronchiolization and increases expression of TGF-β in the lungs following bleomycin administration

Finally we examined whether the lowered catalase activity in the lungs would worsen lung fibrosis induced by bleomycin. The lungs of untreated acatalasemic mice appeared morphologically normal, and no fibrosis was observed at the level of light microscopy, as previously described [35].

**Figure 6:**
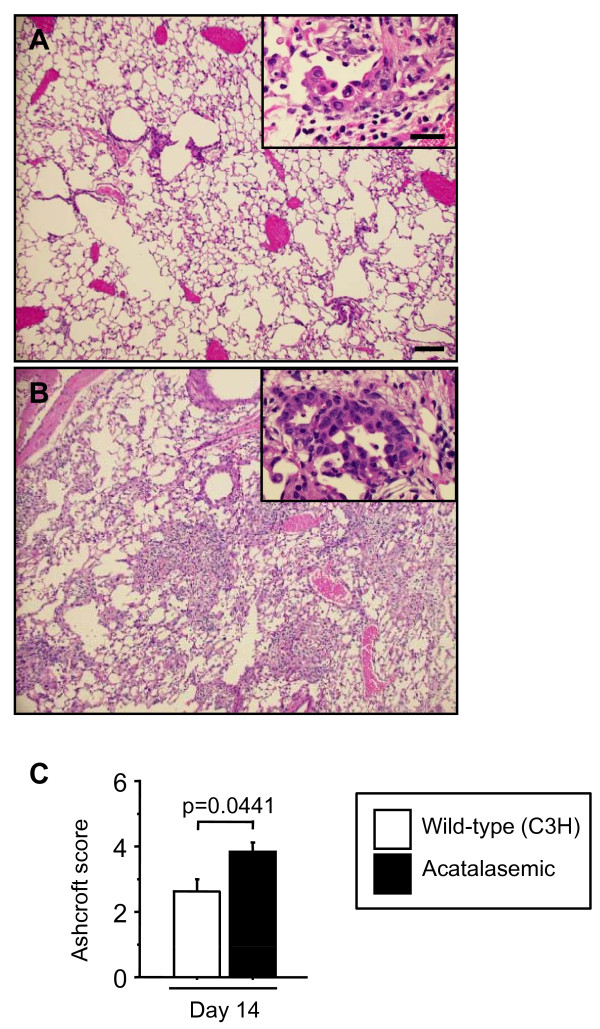
**Lung histology with hematoxylin and eosin staining and Ashcroft score at 14 days**. Wild-type mice demonstrates only mild fibrosis (A), whereas acatalasemic mice shows more severe fibrosis along with infiltration of inflammatory cells in the lungs (B). In fibrotic regions of the lungs, clusters of cuboidal bronchiolar-appearing epithelium were present adjacent to bronchioles ("bronchiolization") in each inset, a cluster of single-layered cells (grade I) in A and tubular structures of stratified cuboidal cells (grade II) in B. (C) Ashcroft score is higher in acatalasemic mice than in wild-type mice at 14 days after bleomycin administration. Scale bar = 100 μm. Scale bar in inset = 25 μm.

**Figure 7:**
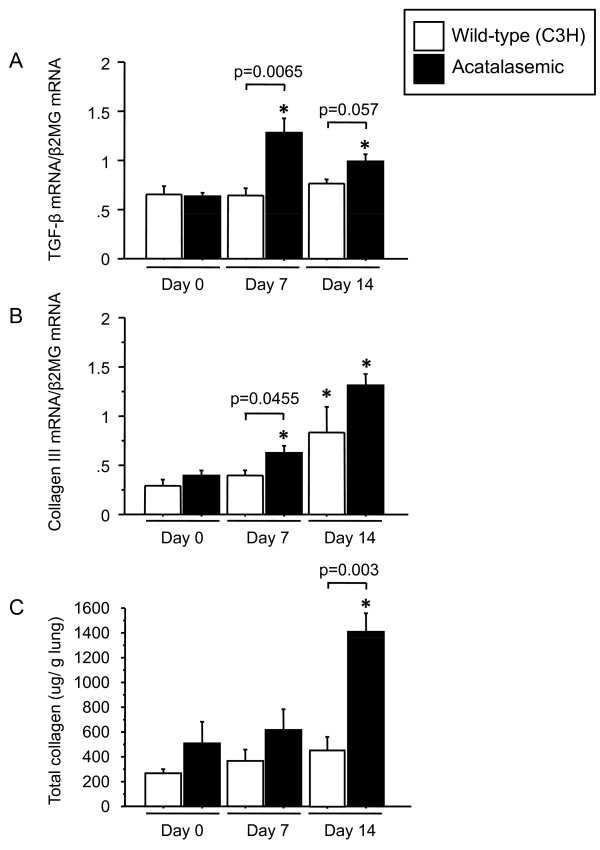
**Changes in expression of TGF-β and collagen in wild-type and acatalasemic mice**. TGF-β mRNA (A), collagen III mRNA (B) and total collagen content (C) of the lungs are higher in acatalasemic mice at 7 and 14 days, at 7 days, and 14 days, respectively. β2MG, β2-microglobulin. *; p < 0.05 vs. Day 0. Day 0; untreated, Day 7; 7 days after bleomycin administration, Day 14; 14 days after bleomycin administration.

**Figure 8:**
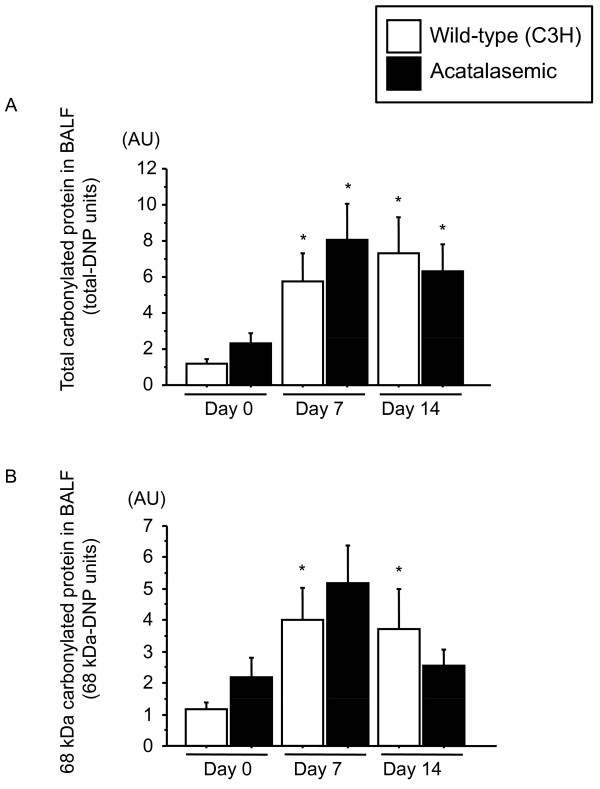
**Changes in expression of carbonylated protein in wild-type and acatalasemic mice**. Total (D) and 68 kDa (E) carbonylated proteins in BALF have no differences between wild-type mice and acatalasemic mice. *; p < 0.05 vs. Day 0. Day 0; untreated, Day 7; 7 days after bleomycin administration, Day 14; 14 days after bleomycin administration.

Fibrosis was more severe and more inflammatory cells were present in the lungs of acatalasemic mice compared with wild-type mice at 14 days after bleomycin administration (Figure 6A, B). Acatalasemic mice demonstrated significantly higher Ashcroft scores at 14 days after bleomycin administration, compared with those of wild-type mice (p = 0.0441) (Figure 6C). Bronchiolization is a metaplastic lesion characterized by cells resembling the lining of the bronchiolar epithelium with normal or thickened alveolar walls, often in acinar formation. It is derived from terminal bronchiolar epithelium through aberrant cell proliferation and migration. It should also be noted that bronchiolization appeared in fibrotic lesions both in wild-type and acatalasemic mice, although the composite bronchiolization score was significantly higher in acatalasemic mice compared to wild-type mice in accordance with the severity of fibrosis (p = 0.0387) (Figure 6A, B, Table 3). Lung fibrosis is characterized by the accumulation of extracellular matrix proteins, such as collagen III. A variety of pro-fibrotic molecules are believed to play roles in the regulation of the fibrogenic process, in which TGF-β is particularly considered to promote fibrosis [4,36]. In our acatalasemic mice, the levels of TGF-β expression were significantly higher in whole lungs at 7 days after bleomycin treatment compared to those of wild-type mice (p = 0.0065) (Figure 7A). Collagen III expression was higher in acatalasemic mice at 7 days and total lung collagen was also significantly elevated at 14 days after bleomycin administration compared to wild-type mice (p = 0.0455 and p = 0.003, respectively), suggesting accelerated fibrinogenesis in the acatalasemic mice (Figure 7B and 7C). To assess whether acatalasemic mice exhibit excessive oxidative stress in the lungs after bleomycin administration, we examined BALF protein carbonyls, an oxidative stress marker, at 0, 7 and 14 days. Bleomycin induced the increase of total carbonylated protein and 68 kDa carbonylated protein both in wild-type and acatalasemic mice. Acatalasemic mice showed modest further increases at 0 and 7 days, but the differences did not reach the statistical significance between wild-type and acatalasemic mice (Figure 8A and 8B).

**Table 3:** Incidence of bronchiolization in the lung

	Grade 1	Grade 2	Grade 3	composite bronchiolization score
Wild-type (C3H) (n = 8)	2.9 ± 1.0	2.0 ± 0.8	3.0 ± 1.3	13.4 ± 4.0
Acatalasemic (n = 6)	6.7 ± 2.0	2.0 ± 0.7	3.0 ± 1.1	28.3 ± 5.0*

*: p < 0.05 vs. Wild-type mice (C3H/AnLCsaCsa) (Mean ± SE)

Grade 1: Single alveolus lined by cuboidal epithelial cells or a single isolated acinar structure consisting of cuboidal epithelial cells adjacent to a terminal bronchiole

Grade 2: 2 to 4 clustered tubular structures consisting of single-layered cuboidal epithelial cells adjacent to a terminal bronchiole

Grade 3: More than 4 clustered tubular structures single-layered or stratified cuboidal epithelial cells

## Discussion

In human fibrotic lungs, we observed a decrease in catalase activity as well as in mRNA and protein levels. Bronchiolar epithelium is a major site of catalase expression in normal adult lungs. Decrease of catalase in bronchiolar epithelium and in abnormal re-epithelialization suggests the presence of intracellular oxidative stress in those specific cell types in fibrotic lungs. We have been long interested in the role of aberrant proliferation of bronchiolar epithelial cells, such as alveolar bronchiolization and squamous metaplasia, in the pathophysiology of lung fibrosis. Although it appears temporarily in the bleomycin model, the degrees of alveolar bronchiolization and of fibrosis were higher in acatalasemic mice compared to those in wild-type mice. We previously demonstrated that those cells of abnormal re-epithelialization were characterized by diminishment of caveolin-1 and the increased expression of matrix metalloproteinases (MMPs) and extracellular matrix metalloproteinase inducer (EMMPRIN) in lung fibrosis [17,18]. Furthermore, we found that MMP-9 is required for the formation of bronchiolization [20]. A recent report has indicated that MMP-7 (matrilysin-1) mediates the aberrant cell proliferation and migration of bronchiolar epithelial cells, implying potential premalignancy [37]. The interaction of these molecules with catalase has been reported in other cell types. For example, treatment with a catalase/superoxide dismutase mimetic, or adenoviral-mediated overexpression of catalase, inhibits hydrogen peroxide-stimulated EMMPRIN upregulation in cardiac myocytes [38]. In another study detoxification of hydrogen peroxide by administration of catalase resulted in a decrease in the MMP activity and cell proliferation in metastatic tumor cells [39]. Collectively, these findings suggest that the loss of catalase in bronchiolar epithelium is involved in abnormal repair of epithelium in fibrosis directly or indirectly via MMP molecules. It also should be noted that fibroblastic foci were exclusively negative for catalase in fibrotic lungs. Although the origin of fibroblast and fibroblastic foci remains to be clarified, they are not source of catalase in fibrotic lungs.

We next addressed whether lowered catalase activity would subsequently worsen inflammation/fibrosis in the lungs. The acatalasemic mouse strain was established by Feinstein et al. from the progeny of x-ray-irradiated mice [40]. We found in the present study that the lung tissues of acatalasemic mice possess only 8.1% of the catalase activity of wild-type mice, although acatalasemic mice demonstrate equivalent levels of catalase mRNA. This suggests that the mutation does not act at the level of gene transcription or mRNA stability, but rather at the level of mRNA translation and/or protein turnover, as reported for other organs of acatalasemic mice [41]. Taken together with previous reports that acatalasemic mice are susceptible to oxidative renal fibrosis [35,42] and peritoneal fibrosis [43], our data also support that catalase plays a crucial role in protection from fibrotic disorders.

There were a few unexpected findings in this study, namely the prolonged upregulation of proinflammatory cytokines, including neutrophilic chemokines, KC and MIP-2, and subsequent neutrophilic inflammation following bleomycin administration in acatalasemic mice. Our data suggest that a lack of catalase activity potentially enhances the recruitment of neutrophils after bleomycin administration into the lungs. Sustained upregulation of proinflammatory cytokines may, at least partly, explain the elevation in neutrophils even at 14 days after bleomycin administration. Although the cellular source of these proinflammatory cytokines remains to be determined in this model, the upregulation observed may be related to the absence of intracellular catalase activity.

Several limitation of this study should be noted. In the present study, we used bleomycin for the lung fibrosis model. Bleomycin, a clinically important causative agent in lung fibrosis, is widely used in experimental models of human disease resembling pulmonary fibrosis [44]. Although the precise mechanism of bleomycin-induced fibrosis is yet to be determined, alveolar cell damage and subsequent pulmonary inflammation is particularly important in the development of lung fibrosis. Studies of this model have helped uncover the complexity of mechanisms involved in the human disease upon understanding the limitation. First of all, fibrosis eventually resolves in mice 6 to 10 weeks after bleomycin injury [45]. This does not occur in humans. Similar to fibrosis, the decrease in catalase at the levels of activity and mRNA expression was also temporal; catalase levels appeared to increase again at 21 days (Figure 3) and tended to become restored at 35 days before the resolution phase (data not shown). This fact is notably in sharp contrast to the persistent decrease of lung catalase in human IPs (Figure 1). It is also been known that the susceptibility to fibrosis varies among the different strains of mice [46-48]. Compared to C57BL/6J mice, the background strain of acatalasemic mice C3 H is reportedly to be rather resistant to this model [47]. That C3 H wild-type mice did not exhibit apparent lung fibrosis after bleomycin administration is consistent with the findings shown in Figures 6A and 7. The elevation of BALF protein carbonyls after bleomycin administration suggests the presence of extracellular oxidative stress in the lungs, which is consistent to the previous papers [49,50]. However, there is no further oxidative stress in acatalasemic mice as compared to wild type mice. This raises a possibility that the increased fibrosis in acatalasemic mice may not be due to excessive extracellular oxidative stress, although this could be related to an insensitive way of measuring the oxidative stress. Our result that catalase is decreased in bleomycin-induced lung fibrosis might be due to changing cellular dynamics during progression, however, the finding that the acatalasemic mice get more fibrosis supports the functional role of catalase in preventing fibrosis.

Taken together, this study supports the concept that a complicated network consisting of oxidant-antioxidant imbalance and inflammation closely contributes to the progression of fibrosis, in which catalase plays a putative role for protection of the lung.

## List of abbreviations

BAL: bronchoalveolar lavage; BGUS: β-glucuronidase; CXCL1/KC: keratinocyte-derived chemokine; CXCL2/MIP-2: macrophage inflammatory protein-2; EMMPRIN: extracellular matrix metalloproteinase inducer; GAPDH: glyceraldehyde-3-phosphatase-dehydrogenase; IL-1β: interleukin-1β; LCM: laser capture microdissection; β2MG: β2-microglobulin; MMPs: matrix metalloproteinases; NSIP: nonspecific interstitial pneumonia; PCR: polymerase chain reaction; ROS: reactive oxygen species; RT: reverse transcriptase; Sjs: Sjögren's syndrome; TGF-β: Transforming growth factor-β; UIP: usual interstitial pneumonia.

## Competing interests

The authors declare that they have no competing interests.

## Authors' contributions

NO and TB have designed the study and written this paper. NO has mainly performed the experiments. DHW, TT and KO have provided the acalasemic mice. KN, CM and MN supervised the research. This manuscript has been read and approved by all authors.
